# Clinical and Evolutionary Features of SARS-CoV-2 Infection (COVID-19) in Children, a Romanian Perspective

**DOI:** 10.3390/children9091282

**Published:** 2022-08-25

**Authors:** Gheorghiță Jugulete, Daniela Pacurar, Mirela Luminița Pavelescu, Mihaela Safta, Elena Gheorghe, Bianca Borcoș, Carmen Pavelescu, Mihaela Oros, Mădălina Merișescu

**Affiliations:** 1 Faculty of Medicine, University of Medicine and Pharmacy, “Carol Davila”, No. 37, Dionisie Lupu Street, 2nd District, 020021 Bucharest, Romania; 2 “Matei Balş” National Institute for Infectious Diseases, No. 1, Calistrat Grozovici Street, 2nd District, 021105 Bucharest, Romania; 3 Department of Pediatrics, “Grigore Alexandrescu” Emergency Clinical Hospital for Children, No. 30-32, Iancu de Hunedoara Blvd., 011743 Bucharest, Romania; 4 Ponderas Academic Hospital, No. 85A, Nicolae G. Caramfil Street, 014142 Bucharest, Romania; 5 Faculty of Medicine, Titu Maiorescu University, No. 67A, Gheorghe Petraşcu Street, 3rd District, 031593 Bucharest, Romania

**Keywords:** SARS-CoV-2, COVID-19, children, clinical features, comorbidities, male gender

## Abstract

Background: Given the potential for additional development to clarify a better knowledge of generally influence of COVID-19 upon the pediatric population, the clinical symptoms of SARS-CoV-2 infection in children and adolescents are still being explored. Morbidity in children is characterized by a variable clinical course. Our study’s goal was to compare clinical aspects of 230 pediatric patients who analyzed positive for SARS-CoV-2 and were hospitalized between April 2020 and March 2022. Methods: This retrospective study aimed to compare the clinical characteristics of coronavirus disease 2019, (COVID-19) in two groups of pediatric patients hospitalized in the infectious disease clinical ward IX at the National Institute for Infectious Diseases “Prof. Dr. Matei Bals,” Bucharest, Romania. Clinical characteristics of 88 patients (first group), admitted between April–December 2020 were compared with the second group of 142 children admitted between July 2021 and March 2022. Results: Of 230 children, the median age was 4.5 years, and 53.9% were male. Fever (82.17%) and sore throat (66%) were the most common initial symptoms. Rhinorrhea (42%), cough (34%) and diarrhea (41.74%), with abdominal pain (26%) were also reported in a considerable number of cases. 88 (36.21%) patients (first group) were admitted during the second wave in Romania, mostly aged <5 years old, and experienced digestive manifestations like fever (*p* = 0.001), and diarrhea (*p* = 0.004). The second group experienced different clinical signs when compared with the first group, with higher temperature and increased respiratory symptoms analogous to persons who suffer acute respiratory viral infections. The proportion in the second group increased by 23.48% from the first group, and the 0–4 age group for both groups had symptoms for a median interval of 5 days; age (0–4-years old) and length of stay were both proportionally inversely and required longer hospitalization (5 days), for the first group. During study time, the fully vaccinated children for 5–12 years old were 10%, and for 13–18 years old, 14.35% respective. We report two Pediatric Inflammatory Multisystem Syndrome (PIMS) in the second group, with favorable evolution under treatment. Comorbidities (obesity and oncological diseases) were reported in both groups and are risk factors for complications appearing (*p* < 0.001). All pediatric cases admitted to our clinic evolved favorably and no death was recorded. Conclusions: Clinical characteristics of pediatric patients with COVID-19 are age-related. In the first group, 85.29% of 0–4 years old children experienced digestive symptoms, whereas in the second group 83.78% underwent mild and moderate respiratory symptoms for the 5–12 age range. The potential effects of COVID-19 infection in children older than 5 years should encourage caregivers to vaccinate and improve the prognosis among pediatric patients at risk.

## 1. Introduction

The severe acute respiratory syndrome coronavirus 2, SARS-CoV-2, has expanded rapidly since December 2019, making it the greatest epidemic of the twenty-first century [1,2,3,4,5,6,7,8]. Clinical evidence indicates that this disease primarily affects adult populations, bypassing most youngsters who, from the start, have only had modest symptoms [9,10,11,12]. Nevertheless, many features of SARS-CoV-2 infection in children and adolescents remain uncertain [13].

This study intends to describe and compare clinical features of children hospitalized for COVID-19 at the infectious diseases clinical ward IX at the National Institute for Infectious Diseases “Prof. Dr. Matei Bals”, Bucharest, Romania. This information could contribute to a wide understanding of this disease in pediatric practice.

Compared with the adult population, a smaller number of children were admitted to the clinic related to COVID-19 infection [14]. Data from around the nation and the world showed that just 2 to 3 per cent of affected kids needed to be hospitalized [15]. According to evidence, up to 50% of children with coronavirus infection may not show any symptoms and hence go undiagnosed [16,17]. Children’s COVID-19-related mortality makes up about 2% of all deaths [18]. Children are less likely to get severe disease symptoms if they test positive for SARS-CoV-2, according to both scientific and empirical data that has a certain degree of accuracy. Angiotensin-converting enzyme number 2 (ACE-2) expression levels, prior endothelium damage, and innate immunity are the three main factors that a recent study is intending to correlate to explain this shift. As an age-related condition, higher levels of ACE-2 expression in children’s alveolar epithelium have been linked to the beginning of acute respiratory distress that may occur after septic infection or other respiratory diseases which are not due to COVID-19, among other illnesses or conditions [19,20,21].

A knowledge gap exists concerning children’s low sensitivity to COVID-19 and the emergence of mild disease in the pediatric population. Fever, coughing, nausea, vomiting, diarrhoea, skin rashes, anosmia, fatigue, headaches, muscle stiffness, shivers, and nasal congestion are common manifestations. Pediatric comorbidities, such as diabetes, asthma, heart congenital disorders, central nervous system diseases, asthma, and metabolic diseases, raise the probability of hospitalisation to critical care unit, but it is typically still low for children compared with adults [22].

The family Coronaviridae includes enveloped RNA viruses known as coronaviruses (CoVs). SARS-CoV-2 is a unique homologous strain of SARS-CoV-1, triggering the largest CoV pandemic outbreak known as coronavirus illness 2019 started in December 2019 in Wuhan, China (COVID-19). The three outbreaks that have been associated with CoVs in recent years have spread quickly, demonstrating their capacity to transcend species-specific barriers and cause epidemic and pandemic diseases that have a significant negative impact on human health.

An important factor influencing the extent of SARS-CoV-2-host contact, tissue tropism, and viral pathogenesis is the binding between the S glycoprotein and the human cellular receptor angiotensin-converting enzyme 2 (hACE2). The first step of a virus’ entrance into cells is this binding [23]. SARS-CoV-2 has a strong potential for transmission from person to person due to the high affinity with the S glycoprotein of the virus binds with the hACE2 receptor in humans. Additionally, it has been noted that SARS-CoV-2 S glycoprotein has a higher binding affinity to the hACE2 receptor than SARS-CoV-1. This strong binding potential presumably denotes an interspecies affinity. hACE2 is found throughout the body in several organs, including the kidneys, heart, testicles, and lungs. It is expressed in human epithelial alveolar cells in the respiratory tract, where it promotes SARS-CoV-2 infection [23,24,25].

The pandemic evolved in successive waves (6 so far) without a specific season but with a predominant specific strain for each of them (strains α, β, γ, μ, δ, omicron). The coronaviruses HCoV-NL63, HCoV-OC43, HCoV-229E, and HCoV-HKU1 are the four most prevalent ones. According to a Chinese study by Zeng ZQ, Chen DH, Tan WP, et al., hospitalized children with acute respiratory tract infections had a 4.3 per cent positive rate for these four HCoV infections [26]. Common human coronaviruses with its 4 serotypes: 229E, NL63, OC43 and HKU1, cause in immunocompetent individuals light or moderate infections of the upper respiratory tract, clinically manifested by fever, altered general state, rhinorrhoea, odynophagia, cough, headache). In immunocompromised persons and those of extreme ages, coronaviruses can determine lower respiratory tract infections such as pneumonia or bronchitis. Most people get infected with one or several of these viruses at some point in their lives thus the adult population presents serotype protective neutralizing antibodies [26,27,28,29,30].

Based on the clinical characteristics, laboratory results, and chest radiograph imaging, including asymptomatic infection, the severity of COVID-19 was classified in four different groups, from asymptomatic to critical. The following severity score was used for the diagnostic standards in our study groups. (See Table 1) [31].

In Romania, the first cases of infection with the new coronavirus were registered in March 2020. The diagnoses were made based on the patient’s direct exposure to SARS-CoV-2, clinical symptoms such as cough, fever, and breathing difficulties without a known cause, and confirmation through RT-PCR testing. COVID-19 has various clinical forms, from asymptomatic to severe ones, some with evolution to multiple organ failure and death. Severe clinical forms are frequently met in patients with comorbidities (cardiovascular diseases, diabetes, renal impairment, obesity, tumors). In children with SARS-CoV-2, the infection is usually asymptomatic, or mild but more severe clinical cases have been observed especially in patients with associated risk factors (obesity, type 1 or 2 diabetes, cardiovascular diseases, chronic pulmonary diseases, including asthma, chronic renal diseases, including dialysis, chronic liver diseases, immunosuppression, human deficiencies, HIV/AIDS, prolonged usage of immunosuppressors) [30,34].

One life-threatening complication is Multisystem Inflammatory Syndrome associated with COVID-19 (MIS-C or PIMS-TS) that can occur in SARS-CoV-2 infection in children and adolescents, especially during the recovery time [12].

The diagnostic is established based on the assessment of the presence of the inflammatory syndrome (certified by laboratory investigations) associated with multiple-organ failure (renal, cardiac, haematological, respiratory, gastrointestinal, dermatological, neurological). These cases featured in Literature during the first pandemic waves when severe forms of diseases in children were also registered, with an incidence rate of approximately 1 to 5000 cases of COVID-19 [14,35]. The first cases were documented in USA and Italy, both in previously healthy children as well as in those with comorbidities [36,37,38,39,40,41].

## 2. Material and Method

The authors have undertaken a clinical retrospective study on COVID-19 paediatric cases admitted to National Institute for Infectious Diseases “Prof. Dr. Matei Bals”, Bucharest, Romania between April 2020–March 2022. The following parameters were monitored: age, gender, clinical signs, evolution, complications, and comorbidities. Data were collected from medical records, and all the children included in our study had laboratory-confirmed SARS-CoV-2. Nasopharyngeal swabs were taken from all patients who addressed an acute illness at the National Institute for Infectious Diseases, Bucharest, Romania within 24 h of admission and tested using a reverse transcription-polymerase chain reaction (RT-PCR) kit (Thermo Scientific, Waltham, MA, USA). Viral RNA extraction was performed on the King Fisher Flex (Thermo Fisher, was done using Allplex 2019-nCoV Assay (Seegene, Seoul, Korea) and BioRad, Hercules, CA, USA thermocycler.

**Figure 1:**
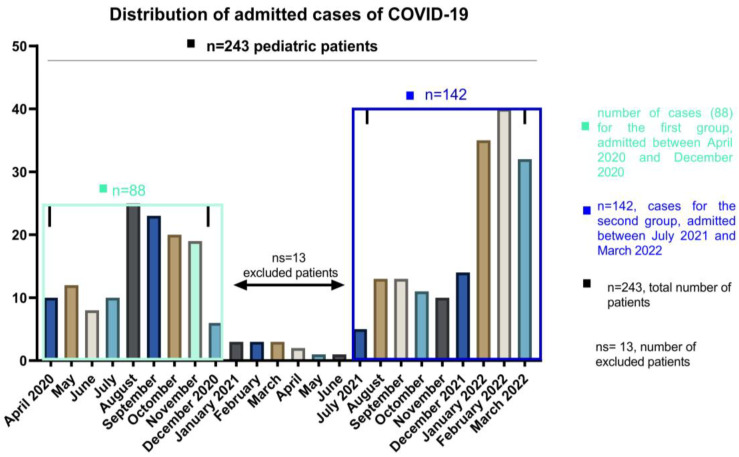
The number of COVID-19 cases of pediatric patients, hospitalized between April 2020 and March 2022.

Patients aged less than 18 years on the date of hospital admission were enrolled on the study group. The patients were divided into 3 different age groups, 0–4 for infants and toddlers, 5–12 for children and 13–18 years old for teens and older. We included the patients according to the distribution of the waves of COVID-19 among pediatric patients in our country, and the exclusion criteria were related to the number and the impossibility of comparing such a small number with the other waves of patients admitted in the periods April–December 2020 and July 2021–March 2022. The 13 patients were therefore excluded based on their number and not on symptomatology, which is similar to the other age and gender categories. Details of demographic data are presented in Table 1. For this report, we included a total of 230 patients who resemble the peak distribution waves at admission as follows: the first group of 88 patients, which corresponds to the year 2020 (between April and December 2020), and the second group of 142 patients which were admitted between July 2021 and March 2022. (See Figure 1). Among 243 pediatric patients, 13 of them who were hospitalized between January and June 2021 were not included in the research group as we used to compare the larger groups of the pandemic waves. When values were regularly distributed, we presented continuous variables as mean (SD: standard deviation) and median (IQR: interquartile range) otherwise. Categorical variables were presented as frequencies and percentages. When comparing groups, categorical data were using and we show results using Chi-square or Fisher’s test, while for continuous data we used Mann–Whitney test and two-way ANOVA to explore the main factors influencing comorbidities. Significant statistical differences resulted in *p* < 0.05. Statistical analyses were performed using GraphPad Prism 9.4.0 (GraphPad, CA, USA).

The study was carried out according to Helsinki Declaration of 1964 and approved by the ethics committees of the National Institute for Infectious Diseases “Prof. Dr. Matei Bals”, Bucharest, Romania. Informed consent was obtained from all parents and legal guardians of patients included in the study.

## 3. Results

During the study period, a total number of 230 pediatric COVID-19 infections were recorded at National Institute for Infectious Diseases “Prof. Dr. Matei Bals”, Bucharest, Romania. All cases of SARS-CoV-2 in children evolved favorably, without deaths through the study, but two cases (from the second group) presented Multiple Organ System Failure with favorable evolution under treatment. Figure 1 evidence the distribution in waves of admissions, with a peak registered between January and March 2022. The gradual increase in the number of cases was the consequence of a highly contagious strain selection.

The analysis of SARS-CoV-2 associated deaths showed that in our ward the rate of mortality was zero in children while in adult patients it reached 2.05% (29/1.412) reported as the number of adult patients hospitalized in the same period at our hospital. In terms of demographic characteristics, there were more males in both arms, 55% in the first group and 53.5% in the second group, and have statistics significance, and the mean value for age in the first group was lower compared with those admitted in the second group (0.8 years (IQR: 0.6, 7.9 years) vs. 1.5 years (IQR: 1.5, 12.8 years); *p* < 0.001. (See Table 2).

The length of hospitalization for the first group of pediatric patients was 4 days (IQR 2–5 days) compared with the second group where the average length of hospitalization was 5 days (IQR 3–7 days), though with a statistical significance of *p* = 0.04. The hospitalization rate was higher among children between 0–4 years old, for both groups (*p* = 0.01). Bold values show statistical significance.

In what concerns the onset clinical picture of SARS-CoV-2 in children it has been observed to be polymorphous and particularized based on the child’s age. Thus, in small children (under the age of 1, both groups) digestive symptomatology predominated as lack of appetite, and diarrhea (*p* < 0.0001), acute dehydration syndrome (without statistical significance) accompanied by fever which was documented in almost all patients-83% vs. 81.9%; (see Table 2).

**Figure 2:**
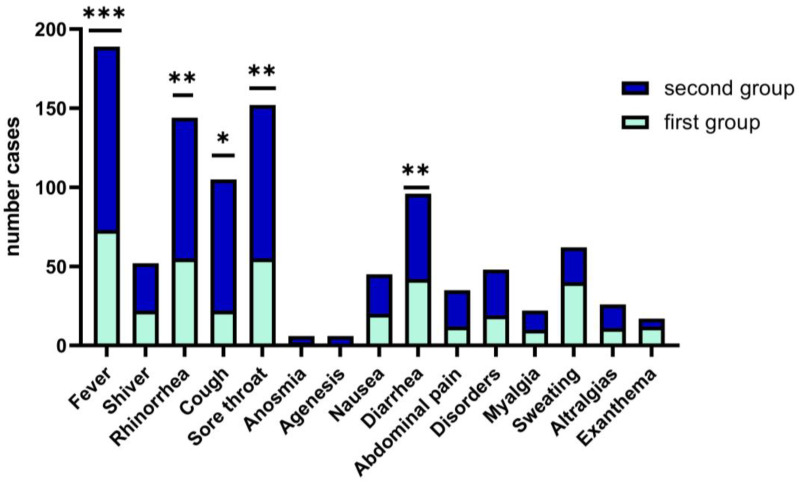
Wilcoxon matched-pairs signed rank test. Clinical features of COVID-19 in children between the two groups. Data are represented as the number frequency of the symptoms in each group, *p*-value (two tailed, was significantly effective, *—*p* ≤ 0.05, **—*p* ≤ 0.01, ***—*p* ≤ 0.001).

In children and teens age groups, the clinical picture of upper respiratory tract infections such as fever, rhinorrhea, odynophagia, dysphagia, cough, and sore throat (68%) is polymorphous with the occurrence of respiratory distress (62.68%) and fever (81.9%), and required longer hospitalization, median days (5), with statistics significance (*p* = 0.04). We have encountered rarely systemic type manifestations in children (exanthema, arthralgias, headache, agenesis and anosmia that are specific to the adult patient with COVID-19 (Figure 2).

**Figure 3:**
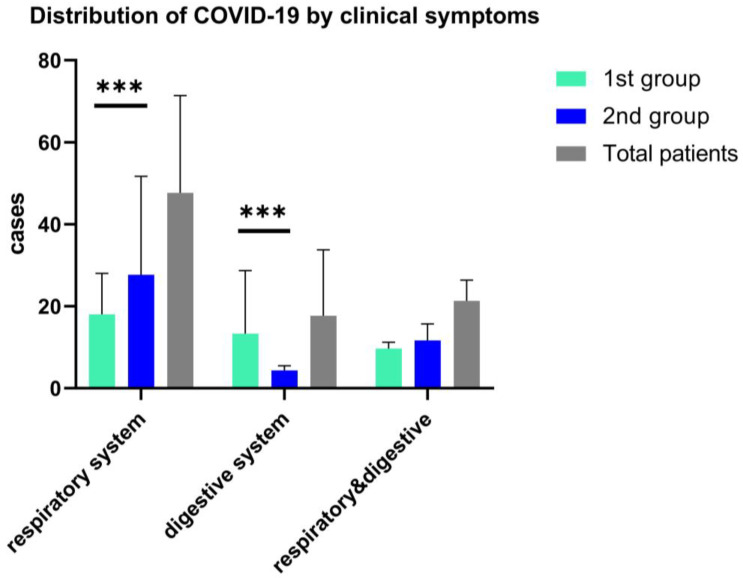
Distribution of COVID-19 in children by the onset clinical features, ***—*p* ≤ 0.001. Data are represented as mean with SD and shows the differences of respiratory and digestive symptoms and both clinical manifestations in a two-way ANOVA statistical analysis.

Clinical symptoms in both study groups associated fever (>38.5 °C), with cough and sore throat more frequently observed in the second group, 68% vs. 62%, *p* = 0.05), while for the first group of children, fever was present with the digestive tract clinical manifestations, and 47.73% associated diarrhea. (See Figure 3). Other less frequent clinical features are respiratory symptoms associated with gastrointestinal symptoms, as we found in both arms, without statistical significance (18.3% vs. 17.9%); see Figure 3.

**Figure 4:**
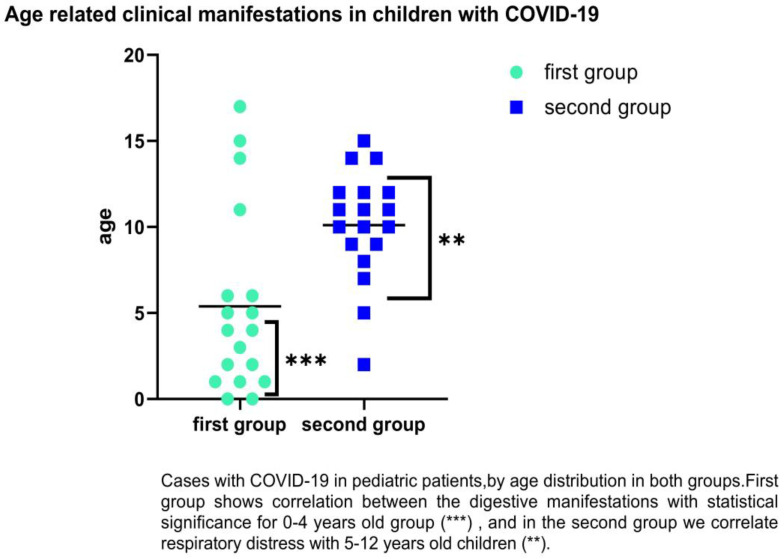
Distribution of COVID-19 cases in children by age and clinical symptoms (respiratory and digestive). Statistically significance **—*p* ≤ 0.01, ***—*p* ≤ 0.001.

We followed the groups by age-related category, and it was observed that SARS-CoV-2 infection in children of first group predominates in the age groups of 0–4 years old with gastrointestinal symptoms such as diarrhea, abdominal pain, or vomiting (38.64% vs. 26.76%). For the second group, in the category of 5–12 years, we found (38.64% vs. 52.11%) with respiratory distress. There were significant differences for 0–1 years old, which encountered 42.86% diarrhea (*p* = 0.0001), and 5–12-years-old (*p* = 0.02) for respiratory distress as the main clinical symptoms (83.78%); (See Figure 4).

**Figure 5:**
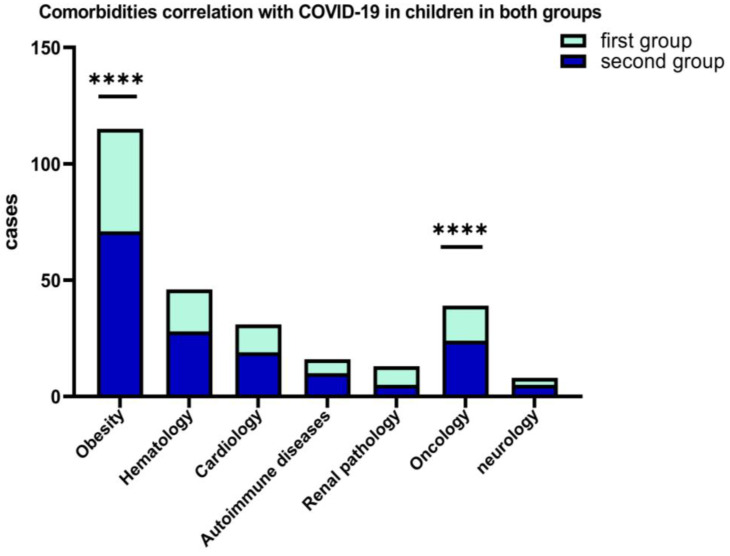
Results of 2-way ANOVA to compare the main effects of the associated COVID-19 comorbidities (obesity and oncological diseases, ****) in children in both groups.

A percentage of 10.29% of the patients from the first group and 12.45% of the second group, presented various comorbidities at the time of hospitalization (obesity, chronic hematological, metabolic, neurological, autoimmune, and oncological diseases). Figure 5 shows that, among the associated comorbidities of COVID-19 in children, the best represented were obesity for both groups, hematological diseases, and oncological pathology.

From the standpoint of clinical forms of COVID-19, it was found that most cases in children were mild and moderate, and it is associated with the presence of comorbidities such as obesity, (*p* = 0.0001) and oncological diseases (*p* < 0.0001). We haven’t registered critical forms of COVID-19 in children but in two situations it was complicated by PIMS in the second group (1.46%).

BMI percentiles were calculated using BMI (kg/m^2^), age, and gender. We used CDC Growth Charts to find the overweight and obesity categories and we associated children with BMI percentiles ≥95th as obesity.

**Figure 6:**
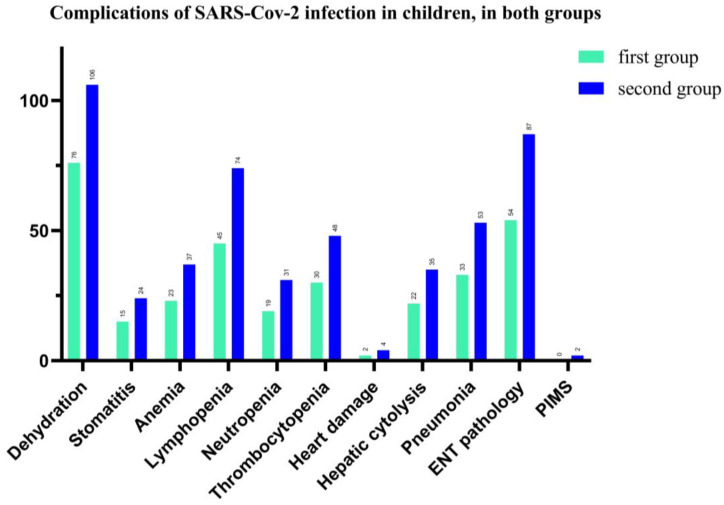
Number of cases with complications of SARS-CoV-2 infection in hospitalized pediatric patients.

In our study, we found that complications associated with SARS-CoV-2 infection in children were represented by 79.13% (both groups) with dehydration, (75 vs. 106 patients), followed by 61.3% ENT pathology, such as pharyngitis, laryngitis, otitis, (54 vs. 87 pediatric patients), and 51.74% for lymphopenia (45 vs. 74 patients). Hepatic cytolysis syndrome has shown in 24.8% cases in total and 22 vs. 35 patients in each group of our study. The hematological picture shows anemia in 26%, neutropenia—21.74% and thrombocytopenia—34% for total patients. We also recorded a significant percentage of cases with acute interstitial pneumonia (37.4%), and 33 vs. 53 children. Cardiac impairment (myocarditis, pericarditis, heart rhythm disorders) was present in 2.4% of cases and 2 children have Pediatric Inflammatory Multisystem Syndrome (PIMS), with a favorable evolution under the established treatment (See Figure 6).

According to the severity classification described by Parri, adapted by Dong et al., (see Table 1), we reported 51% vs. 44.6% mild forms, 59% vs. 65.4% moderate forms, and 0 critical forms.

Of all the patients with mild and moderate forms, 10% received low-flow oxygen via nasal cannula and 16% high-flow oxygen via nasal cannula. Antiviral medication (Remdesivir) was administrated for 19% of children with moderate form and 14% for mild forms without adverse reactions. All hospitalized patients received symptomatic treatment (e.g., fever reduction, cough relief, liver protection, diarrheic relief, related to symptoms). Glucocorticoids (0.4 mg per kg per day) were administrated in 11% of moderate forms and 9% of mild forms, and both cases of PIMS.

In terms of vaccination, we followed the fully vaccinated patients, for the possible categories. For children between 5–12 years old, we report 10% and for teenagers between 13–18 years old, we have a percentage of 14.37%.

## 4. Discussion

In this retrospective study, we compared the clinical features of hospitalized pediatric patients who tested positive for COVID-19, during two waves. Our analysis has shown that in the second group, the average number of cases was increased compared with the first wave analyzed, with older age, a predilection for upper respiratory symptoms, and higher temperature as distinct clinical characteristics. The second group experienced an increase in the number of hospitalization and duration, which is consistent with other systematic reviews [42,43,44]. The first group experienced digestive symptoms, affecting the case severity in hospitalized patients with COVID-19 in age 0–4 years old. Among clinical courses in both groups, the negative prediction was the presence of fever, male gender, chronic condition, and comorbidities, such as obesity and oncological manifestations. For our study, the presence of comorbidities was the reason of longer hospitalization and number of cases especially in the second group, and this observation was not confirmed in other study [43,44]. The disease’s progression varied in our research population, from moderate to mild forms, and no critical to severe patterns were found in each group. The differences in clinical features may indicate that a new variant of COVID-19 can lead to changes in the clinical profile of pediatric patients, especially for categories aged >5 years old.

Assessing the admitted cases and correlating them with other studies, we concluded that slightly more frequent respiratory symptoms children presented respiratory symptomatology in the second group, where the older category was observed [44].

We found a lower percentage of associated clinical manifestations for both digestive and respiratory pathology, as we discovered that in the first group, patients with gastrointestinal pathology had considerably lower ages compared with those in second group by examining the median ages for which they were admitted. Characteristics which significantly influenced COVID-19 infection while the presence of a clinical picture characteristic for upper respiratory tract infections (sore throat, nasal obstruction, rhinorrhoea, cough, odynophagia, dysphagia, dysphonia) sometimes accompanied by difficulty in breathing) was the clinical manifestations of both groups in the 5–12-years old age category. As we followed a retrospective study, it was not feasible to determine the efficiency of antiviral therapy from the current data, but we did not encounter adverse reactions in children.

The main symptom was fever, which was followed by cough, rhinitis, and digestive issues like similar reports [22,45], while other reports show fever and digestive disturbance to appear less common (36–56%) [46]. The reduced size of our study may be a contributing factor to the disparity between the other findings.

SARS-CoV-2 infection in children is more frequent in ages under 5 as vaccinations are absent at this age in the first group. Pediatric COVID-19 cases presented a large picture of complications, the recurring ones being the digestive ones-dehydration, ENT and hematological. A low percentage of children presented cardiac complications and two had PIMS with favorable evolution [22].

Hospital stays were a little bit longer in the second group, which was likely due to complications and comorbidities, especially for obesity pathology, which is similar to those reported in other studies [47]. Obesity comorbid problems have been related to many COVID-19 outcomes. Patients with oncological disorders were also more likely to have increased severity of COVID-19. According to literature and past study, diabetes and heart disease are the two most important risk factors for mortality in patients who have COVID-19, but in our study, we report that the number of children with diabetes and heart disease who are diagnosed with COVID-19 was less frequent, and mortality zero.

Since the vaccination of children and adolescents is strongly influenced by the decision of the parents, we analyzed it from the perspective of general vaccination, given that the vaccination of children aged 5–12 years was started later. There are small percentages to be able to find a statistical correlation in our study, but we intend to follow these developments and show results in the future.

The main limitation of our study is its monocentric nature and the small number of cases included. However, the importance of our reports is the specificity of clinical features in the pediatric population compared with previous studies described before and from the Romanian perspective.

## 5. Conclusions

Admitting that the number of COVID-19 cases in children was not particularly high (230 pediatric patients), there was an increased number of hospitalized children between 5–12 years old in the second group with predominancy of respiratory manifestations, so we can report the evolution of clinical symptoms has emerged in this category. The progression of clinical symptoms and hospitalizations in the older group draws attention to the necessity of vaccination in this category.

The introduction, on a large scale, of COVID-19 vaccination in all age groups represents one of the most effective methods of specific prophylaxis along with non-specific methods (hand washing, physical distancing, protection mask).

Subsequent studies, based on acquired clinical experience, will establish the particularities of SARS-CoV-2 infection in children, useful for identifying the most successful methods of diagnosis and treatment of COVID-19.

## Figures and Tables

**Table 1:** Group classification based on characteristics described by Parri and adapted by Dong et al. [31,32,33].

group classification BY STAGE	Characteristics
Asymptomatic cases	All the following requirements need to be met: chest X-ray (CXR) negative, Absence of symptoms/signs no additional instances meet the criteria, for other groups
Mild cases	Any of the following: symptoms of upper respiratory tract infection or absence of pneumonic focus on CXR. not fitting into another group
Moderate cases	All the following: Cough, signs of severe respiratory distress, and Oxygen < 92% Presence of pneumonia at CXR Absence of criteria for critical group classification Signs of severe respiratory distress/need for any respiratory support
Critical cases	Any of the following: Hospitalisation in ICU/patient intubated/multiorgan failure shock, encephalopathy, renal, cardiac or coagulation dysfunction.

**Table 2:** Epidemiological and clinical characteristics of pediatric patients, between the 1st and 2nd groups and in different age ranges (0–1, 0–4, 5–12, 13–18 years of age). Data are shown as numbers, n (%), and *p*-values were calculated for both groups and were compared using the Mann-Whitney U test. A two-sided *p*-value < 0.05 was considered significant.

Characteristics	First Group	Second Group	*p*-Value
	n (%)	n (%)	
Pediatric patients	88 (38.26)	142 (61.74)	
Male gender (%)	49 (55)	76 (53.5)	0.05
Age-years	n (%)	n (%)	
0–4	34 (38.64)	38(26.76)	**0.001**
5–12	34 (38.64)	**74 (52.11)**	**0.02**
13–18	20 (22.72)	30 (21.13)	0.65
Distress	n (%)	n (%)	
Fever Age range	73 (83)	116 (81.9)	**0.001**
0–1	**23 (31.5)**	10 (8.62)	**0.001**
0–4	20 (27.4)	13 (11.2)	
5–12	18 (24.7)	**68 (58.6)**	**0.001**
13–18	12 (16.4)	25 (21.6)	
Shortness of breath/respiratory distress	55 (62)	89 (62.68)	**0.02**
0–4	18 (32.7)	17 (19.1)	
5–12	12 (35.29)	**62 (83.78)**	**0.04**
13–18	25 (45.45)	**10 (11.24)**	
Sore throat Age range	55 (62%)	97 (68%)	**0.05**
0–4	10 (18.18)	12 (12.37)	
5–12	13(23.64)	**43 (44.3)**	**0.05**
13–18	32(58.2)	**42 (43.4)**	**0.05**
Diarrhoea Age range	**42 (47.73)**	**54 (38)**	**0.0001**
0–1	**18 (42.86)**	12 (22.2)	**0.0001**
1–4	**10 (23.8)**	15 (14.8)	**0.04**
5–12	9 (21.43)	14 (25.93)	
13–18	5 (11.9)	13 (24.1)	
	Days median, [IQR]	Days median, [IQR]	
Hospitalization Age range	**4 [2–5]**	**5 [3–7]**	**0.04**
0–4	**5 [3–5]**	**5 [4–7]**	**0.01**
5–12	4 [3,4]	5 [3–7]	**0.3**
13–18	3 [2–4]	3 [2–4]	
Fully vaccinated, years age	**n (%)**	**n (%)**	
5–12	9 (10.23)	14 (9.85)	0.65
13–18	12 (13.63)	21 (14.79)	0.5

## Data Availability

Not applicable.
